# City Scale Particulate Matter Monitoring Using LoRaWAN Based Air Quality IoT Devices

**DOI:** 10.3390/s19010209

**Published:** 2019-01-08

**Authors:** Steven J. Johnston, Philip J. Basford, Florentin M. J. Bulot, Mihaela Apetroaie-Cristea, Natasha H. C. Easton, Charlie Davenport, Gavin L. Foster, Matthew Loxham, Andrew K. R. Morris, Simon J. Cox

**Affiliations:** 1 Faculty of Engineering and Physical Sciences, University of Southampton, Southampton SO16 7QF, UK; P.J.Basford@soton.ac.uk (P.J.B.); F.Bulot@soton.ac.uk (F.M.J.B.); mac1g12@soton.ac.uk (M.A.-C.); cd3g16@soton.ac.uk (C.D.); S.J.Cox@soton.ac.uk (S.J.C.); 2 Faculty of Environmental and Life Sciences, University of Southampton, Southampton SO17 1BJ, UK; nhcs1g13@soton.ac.uk (N.H.C.E.); Gavin.Foster@noc.soton.ac.uk (G.L.F.); 3 Faculty of Medicine, University of Southampton, Southampton SO17 1BJ, UK; M.Loxham@soton.ac.uk; 4 National Oceanography Centre, Southampton, Southampton SO14 3ZH, UK; andmor@noc.ac.uk

**Keywords:** Internet of Things, wireless sensor networks, air quality, LoRaWAN, Raspberry Pi, urban pollution

## Abstract

Air Quality (AQ) is a very topical issue for many cities and has a direct impact on citizen health. The AQ of a large UK city is being investigated using low-cost Particulate Matter (PM) sensors, and the results obtained by these sensors have been compared with government operated AQ stations. In the first pilot deployment, six AQ Internet of Things (IoT) devices have been designed and built, each with four different low-cost PM sensors, and they have been deployed at two locations within the city. These devices are equipped with LoRaWAN wireless network transceivers to test city scale Low-Power Wide Area Network (LPWAN) coverage. The study concludes that (i) the physical device developed can operate at a city scale; (ii) some low-cost PM sensors are viable for monitoring AQ and for detecting PM trends; (iii) LoRaWAN is suitable for city scale sensor coverage where connectivity is an issue. Based on the findings from this first pilot project, a larger LoRaWAN enabled AQ sensor network is being deployed across the city of Southampton in the UK.

## 1. Introduction

Six Air Quality (AQ) Internet of Things (IoT) devices for monitoring AQ have been deployed across at schools in Southampton, UK [1]. This deployment was a pilot and is currently being expanded to include more sites across the city and surrounding area. The objective was to demonstrate the capability of the AQ IoT devices to capture spatio-temporal variations of Particulate Matter (PM) air pollutants in order to raise public awareness on AQ issues. These devices also acted as a feasibility study for a Low-Power Wide Area Network (LPWAN) technology called LoRaWAN which promises long-range wireless communication to enable sensor deployments in remote areas or locations without connectivity.

Existing AQ sensing networks use a range of sensors to monitor various air pollutants but tend to only have one PM sensor and do not use Low-Power networks (CAIRSENSE [2], Citi-Sense-MOB [3] or EUNetAir [4]). The hardware used can also be considerably more expensive (OpenSense [5], Citi-Sense/Citi-Sense-MOB [6]) than the sensors themselves. This publication shows PM sensor data collected from the AQ IoT devices over a seven-month period, correlated with reference AQ stations within the city. The findings from this pilot deployment are encouraging and show the design and validation of a platform that enables low-cost PM sensors to be deployed simultaneously with long-range wireless technologies for remote data access. The pilot platform is well suited low-cost deployments and permits scaling to a city wide network.

## 2. Air Quality Monitoring

Air pollution has been identified as having a major influence on health worldwide [7], globally 6.5 million premature deaths in 2015 were associated with air pollution [8]. The impact of air pollution levels on health are dependent on pollutant concentrations and exposure levels. These factors vary at fine spatio-temporal scales in urban environments [9,10,11], driving the need for more data by increasing the number of sensor deployments and improving the frequency of sampling.

PM with an aerodynamic diameter lower than 10 μm is of particular relevance and has an impact on health, although PM with an aerodynamic diameter lower than 2.5 μm (PM${}_{2.5}$) has the most strongly linked health effects [12]. PM can cause a wide range of adverse effects on humans even at low concentrations [12]. In the UK, exposure to PM${}_{2.5}$ is responsible for 29,000 deaths per year with an uncertainty of ±75% [7]. The impact of air pollution exposure on health is dependent not only on pollutant concentrations, but also the duration and frequency of the exposure [13], giving rise to a wide confidence interval. At the individual level, various other parameters, including age and health status, also play significant roles [14].

There is an urgent need to collect more data in major urban areas. The pilot deployment focuses on schools as children are one of the most at risk groups [15] and schools are evenly distributed across the city. There is currently a poor ability to determine personal exposure to pollution, given the lack of pollution monitoring stations, and the fine spatio-temporal resolution of pollution variation. High spatio-temporal coverage of pollutants measurements is required to improve the understanding of air pollutant sources and exposure.

At a national level in the UK, PM levels are monitored by the Automatic Urban and Rural Network (AURN) stations [16]. These stations provide reliable and robust data about background concentration levels but are expensive, and require significant expertise to maintain. This makes it difficult to attain the high spatial resolution [17] required to better assess personal exposure and to precisely identify pollution sources. It is therefore clear that different approaches need to be combined to solve this issue [18].

Several innovative air monitoring network projects have been established in cities across Europe and the USA, using low-cost sensors; some use mobile measurements mounted on cars [9], trams [19], bikes [3,20] and pedestrians [21]. Although low-cost sensors offer a means to increase the granularity of the data available, the extent to which they can provide valid data first needs to be evaluated. Once this is qualified, low-cost sensors may be used to complement existing air pollution monitoring networks by providing the spatio-temporal resolution required to improve the understanding of air pollutants and of personal exposure to them [22].

The deployment of a dense, accurate, reliable city-wide network of PM sensors would improve the ability to identify sources of pollution, understand personal exposure, complement existing monitoring networks and raise AQ awareness among the population.

## 3. Particulate Matter Sensors

**Figure 1:**
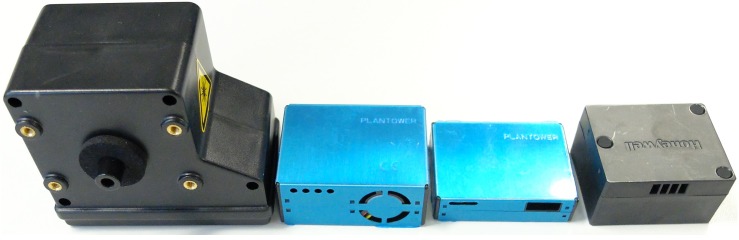
Particulate Matter (PM) sensors: Alphasense OPC-N2 [24], Plantower PMS5003 [25], Plantower PMS7003 [26], Honeywell HPMA115S0 [27].

Figure 1 shows a few low-cost PM sensors which are commonly utilised in air pollution monitors. Their prices range from a few USD to a few hundred USD making it possible to deploy dense city-wide networks. These sensors need to be plugged into a processor (e.g., Raspberry Pi) and equipped with the means to communicate (e.g., WiFi, Bluetooth, LP Networks) and/or store the data collected (e.g., SD Card, Hard drive, flash memory). The most common low-cost PM sensors are Optical Particle Counters (OPCs), based on light-scattering. They can typically detect particles with aerodynamic diameters ranging from 0.3 μm to 10 μm [23]. Below 0.3 μm, the particles do not scatter light sufficiently and, over 10 μm, depending on the actual size of the inlet, they cannot enter the sensor. These sensors transform the signal measured into a raw particulate count and/or mass concentration.

The lower limit of detection of these sensors is generally between 1 and 10 μg/m^3^, which is the same order of magnitude as the World Health Organisation (WHO) annual mean guideline for PM${}_{2.5}$ of 10 μg/m^3^ [28].

Reference methods for measuring PM mass concentration rely on the direct determination of particle mass, rather than inference of particle mass from particle count. Mass concentration is used in the legislation and is necessary for comparing with reference measurement instruments, but the raw particulate count is also useful. For instance, the raw particulate count often offers better data resolution especially when operating close to the sensor’s limit of detection.

The main drawback of using low-cost PM sensors is the data quality, which may be susceptible to: (i) drift over time [2,29], (ii) interference from climate conditions [30], (iii) environmental conditions [31], (iv) a lack of reproducibility between sensor units [32] and (v) the composition of the PM [32]. This susceptibility may vary across different PM sensor models, presenting different characteristics and data quality. In order to address these issues through the pilot project, multiple PM sensors were set-up to run simultaneously alongside one another. Four PM sensors were housed within each AQ device, each sensor was selected based on their popularity in the literature, their ease of use and availability: (i) Alphasense OPC-N2 [24], (ii) Plantower PMS5003 [25], (iii) Plantower PMS7003 [26] and (iv) Honeywell HPMA115S0 [27], as shown in Figure 1; the main characteristics of the sensors are listed in Table 1.

## 4. Air Quality IoT Device

Deploying sensors around a city requires supporting infrastructure and electronics to read, process, store and transmit the sensor data. It is possible to create a network that relies on each sensor or collection of sensors to be a standalone data logger which requires physical access for data retrieval. The ambition of this project was for the devices to become part of a Smart City infrastructure and designed a custom extensible AQ IoT device platform, with sensors, data connectivity and cloud based data storage.

Each AQ IoT device is designed to take advantage of current developments in low-cost technology. The computation is provided by a Raspberry Pi 3 Model B, or Raspberry Pi Hardware Attached on Top (HAT) compatible Single Board Computer (SBC). Connectivity via LPWAN network technologies is becoming common place, despite the very low bandwidth capabilities. Details of competing LPWAN technologies are given in Table 2. This pilot uses LoRaWAN [33] due to its flexibility, ease of use and local coverage. The physical AQ IoT device enclosure and component configuration was developed through an iterative process using rapid-prototyping techniques to evaluate the designs before and during field deployments.

### 4.1. Hardware

The AQ IoT device is built around the Raspberry Pi 3 Model B with a Power Over Ethernet (PoE) [35] HAT stacked on top providing both power and network connectivity. The power is supplied along a Cat5e network cable with a maximum length of 100 m via a standard PoE injector providing a total power budget of up to 15 W. PoE was selected as a power source due to the availability of good quality reliable hardware and progressive IEEE 802.3 standards. The AQ device has a total power draw of 5 W–8 W depending on the number of PM sensors and their configuration. The PoE power supply can be substituted for any other 5 V or 12 V power source, including renewable sources—for example, photovoltaic. It should be noted that the AQ device has been optimised for flexibility and future extensibility rather than power consumption.

A LoRaWAN HAT is stacked on top of the Raspberry Pi and PoE HAT. This long-range wireless technology provides a secondary communication channel and is discussed further in Section 5. The LoRaWAN HAT contains a Global Positioning System (GPS) receiver which is connected to the on-board serial port of the Raspberry Pi. It is currently only used to set the system time Real Time Clock (RTC), but can be utilised in future mobile applications. Temperature and relative humidity may interfere with the readings from some PM sensors [36,37], so a dedicated DHT22 temperature and humidity sensor is located at the main air intake. In the deployed AQ IoT devices, a total of four PM sensors were then attached and contained within an enclosure. Table 3 shows a full list of the hardware included.

Three of the PM sensors require a serial port and the LoRaWAN HAT uses the Raspberry Pi’s single serial port for the GPS device. The additional serial ports were added by using FTDI USB-serial converters because this specific brand supports a unique serial number. The PM sensors are paired with an FTDI USB-serial converter and these serial numbers enable consistent naming and sensor identification between reboots. The sensors can be connected in any order and reconfigured in the field, providing the sensor configuration updates are reflected in the configuration file. The Raspberry Pi’s onboard SPI controller is used to interface with the LoRaWAN module and operates as expected. It should be possible to add the OPC-N2 to this interface as well, but repeatable and reliable behaviour could not be achieved with this configuration when tested. A work around was to include a dedicated USB–SPI converter to support the OPC-N2.

The four PM sensors function differently—for example, the HPMA115S0 does not provide access to a raw particulate count, unlike the other three sensors. The time resolution of the OPC-N2 cannot be lower than 2 s, sending commands more frequently creates communication issues which are exacerbated by the length of the SPI communication wires. The HPMA115S0 has a maximum polling rate of every 6 s, the OPC-N2 was also polled at this rate to avoid the SPI issues. The Plantower PMS5003 and PMS7003 maximum data rate alternates between 1 and 3 s depending on the particulate count. The Plantower PMS7003 is connected to the FTDI Chip via a PCB connector board supplied with the sensors.

**Figure 2:**
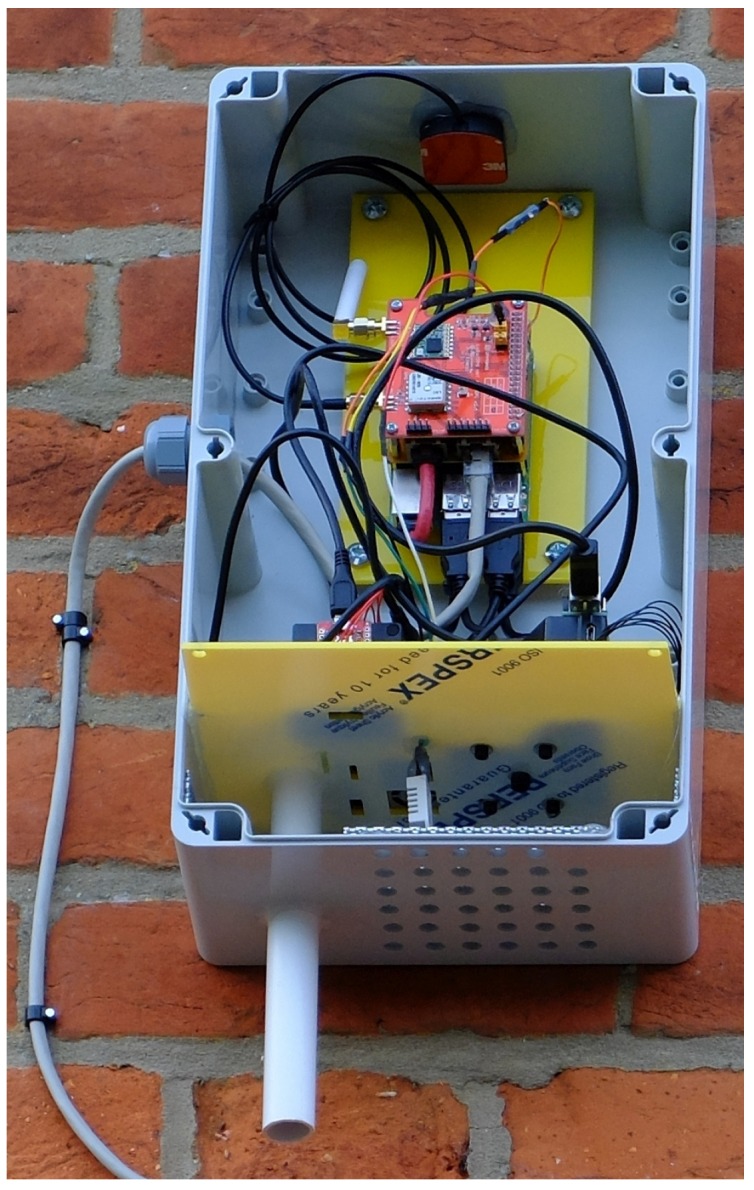
Version 1 of the Air Quality (AQ) Internet of Things (IoT) device, without the enclosure lid; installed on an external wall.

The PM sensors are enclosed in an IP65 ABS enclosure 360 mm × 200 mm × 160 mm (H × W × D). This was mounted in a portrait orientation and grids of 8 mm holes were drilled in the base and side of the enclosure to ensure a constant air flow. To reduce debris and biological material entering the enclosure, these holes were then protected by a 3 mm hole diameter mesh, 1.5 mm thick and 51% open area. The sensors are mounted on a bulkhead inserted across the width of the enclosure as shown in Figure 2. The Raspberry Pi 3 Model B is mounted above the bulkhead in order to provide additional protection from water ingress.

**Figure 3:**
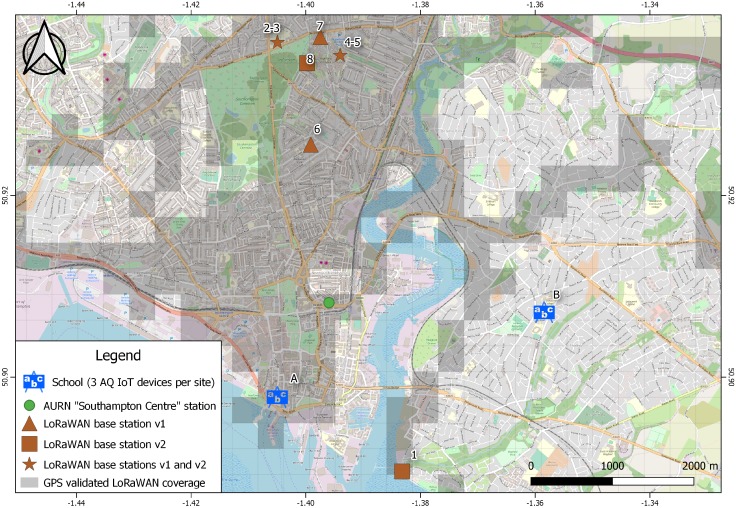
A map showing the six deployed Air Quality (AQ) Internet of Things (IoT) devices at School A & School B; the eight LoRaWAN v1 & v2 base stations; and the GPS confirmed coverage across Southampton, UK [43,44].

The PMS5003 & PMS7003 have the air intake and exhaust on the same side of the sensor. The HPMA115S0 & OPC-N2 exhaust from the opposite side to the air intake; this meant these sensors were exhausting into the main electronic components compartment. To prevent air recirculation and sensor bias, heat produced by the Raspberry Pi is then ducted away from the enclosure, via a 20 mm diameter pipe. Figure 2 shows the complete AQ IoT device deployed on an external wall at School A; see Figure 3 for device location.

### 4.2. Software

When using a Raspberry Pi for remote deployments, careful consideration needs to be given to the Operating System (OS), as some desirable features are not enabled by default. If the OS fails to boot, then physical intervention is required, which can be costly and time consuming. Owing to the pilot deployment time constraints, the latest version of Raspbian Lite for the OS; in parallel, more robust OS was developed. This decision was made to avoid delaying initial hardware deployment, knowing that the Raspbian OS reliability is problematic and needs addressing.

If the Raspbian OS running on a Raspberry Pi is not shut down properly, it can cause file system corruptions and, when running on an unreliable power supply, the probability of this occurring increases. The easiest way to protect against corruption is to use a read-only root file system, as this reduces the likelihood of a write being performed if power is lost. It is these failed writes that can cause file system corruptions. There is still a need for a read/write file system to store sensor data, but preferably kept separate from the OS. This limits file system corruptions to the sensor data file system which can be rectified remotely, as the OS on the read-only file system will always boot the device into a known operational state.

Another issue with the default Raspbian (Stretch) Lite image is the large number of pre-installed packages. This potentially increases the security vulnerabilities, which makes the OS image unnecessary large and increases the number of updates that need deploying. If the device is connected to a high bandwidth network, this is not an issue but is problematic for the bandwidth limited pilot deployment.

In order to address these known Raspbian issues in subsequent deployments, a custom minimal Linux distribution is being built using the Yocto project tools [38]. This route was selected due to the flexibility it offers around choosing only the necessary packages, libraries and binaries. Most of the popular SBC OSs, for example Raspbian, are trimmed down versions of PC distributions. These are valuable for beginners and hobbyists but are too large for resources constrained field deployments. Yocto also supports creating OSs with read only root file systems which address the file system corruption issue.

The custom OS has three major components (i) Over-The-Air (OTA) update capabilities (OSTree) [39], (ii) containerisation (Docker) [40] and (iii) the tools required for the management infrastructure. OTA capabilities allow us to add new packages and make changes to the OS post device deployment, such as security updates. OSTree was chosen because it only sends OS differences as opposed to other tools of this type that send full OS updates needlessly consuming bandwidth, which is often limited in remote locations. OSTree benefits from open source management tools, such as the GENIVI [41], built to ease the management of OTA deployments.

Containerisation allows a minimal base OS to be used as the application container provides all the dependencies needed in order to run. The custom-built OS runs applications in Docker containers, which allows us to remotely add, stop, start and delete applications within containers. One of the most important advantage containerisation offers is ease of building applications independent of the host OS, eliminating dependency problems and enabling portability. It offers fault tolerance, security capabilities, and management tooling such as Cockpit [42], which can manage Docker containers and OS updates. This OS has been developed and tested in parallel to the current deployments that employ Raspbian Lite as the OS.

### 4.3. Deployment

Six AQ IoT devices have been deployed across two school sites within the city of Southampton, as shown in Figure 3. At each school, the deployment positions have been chosen so that the devices are located around the school perimeter, with at least one of the devices being located next to a road influenced by school traffic. School A is the closer of the two sites to the AURN monitoring station “Southampton Centre”. The AQ IoT devices were mounted on exterior walls/fences or railings ≈3 m high. A single Cat5e network cable provides power and network connectivity from an internal source, up to 100 m away.

## 5. Data Connectivity

At the time of publication, the pilot project deployments are live and providing AQ monitoring data. In order to support management and maintenance operations, the ability to connect to the device and transfer data is required. Two main technologies have been investigated: OpenVPN [45] and Secure Shell (SSH) tunnels. The two separate methods of remote access have been chosen to provide resilience, flexibility and to overcome external network configuration changes. Both the OpenVPN and the SSH tunnels connect from the AQ IoT device to a Linux Virtual Private Server (VPS) running on the Microsoft Azure cloud platform. The OpenVPN tunnel allows the AQ IoT device to be accessed directly from the cloud server. The SSH tunnel that the AQ IoT device establishes to the server initiates a reverse tunnel, enabling users on the server to connect into the device.

As the pilot project is extended it is likely not all of the AQ IoT devices will have WiFi or wired network connectivity, so a LoRaWAN module has been included to allow low bandwidth, long range communication [46]; data rates are shown in Table 4. The aim is to validate using LoRaWAN as the sole communication channel. A total of seven base stations have been deployed in Southampton and an 8th has been installed by a 3rd party, as shown in Table 5 and Figure 3.

Table 4 summarises the available data rates for use in LoRaWAN transmissions. As the data rate decreases, the power use increases, as each transmission takes longer. As the data rate decreases, the transmission range increases. After installation of the AQ IoT devices at both schools, it was observed that LoRaWAN messages were not always reliable, requiring changes to the data-rates. It was found that, for this deployment, a Spreading Factor (SF) of 10 gave the best compromise between range and throughput. This is deployment dependent and will need to be re-evaluated as more gateways and AQ IoT devices are added. The pilot LoRaWAN messages are being received at distances over 12 km using SF7, but the coverage is not uniform; over 250,000 GPS validated LoRaWAN data points have been collected from across the city. The tested LoRaWAN coverage is shown in Figure 3 and in the published dataset [47]. Updated coverage is available online [44].

## 6. Air Quality Results

The results of data analysis are presented in this section have been collected over a period of seven months between 8 February and 6 September 2018. The closest AURN monitoring station, “Southampton Centre” [49], is located about 1 km away from School A as shown in Figure 3 and about 2 km away from School B. This station produces hourly PM${}_{2.5}$ concentration data, which is then compared to the hourly PM${}_{2.5}$ concentrations measured by the sensors of the AQ IoT devices. The data produced by this AURN station is readily available from [50]. The data for PM${}_{2.5}$ concentrations from the AURN station were not available for the following periods: 13 February until 7 March, 12 July until 27 July; and 8 August until 13 August. The preliminary analysis of the data collected by the AQ IoT devices in both schools suggest that the four sensors were all able to capture the variations in PM concentrations with variations and patterns similar to the PM concentrations measured by the reference station. In addition, in each school, the variations of PM concentration measured by the sensors of the same model were similar. The HPMA115S0 was initially thought to be faulty or unable to measure low concentrations [1], but it was due to a technical error in the configuration of the AQ IoT device electronics. Due to resources and access, this was only resolved in one of the six locations in this deployment, in School A; future updates will include the modification.

**Figure 4:**
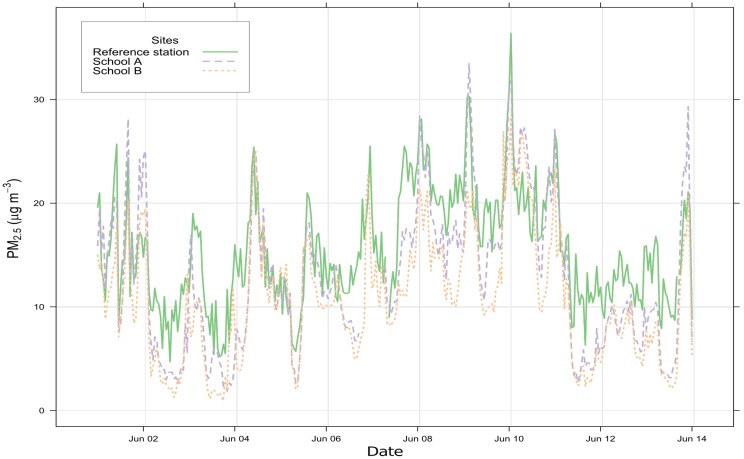
Time series comparing the PM_2.5_ concentrations reported by the “Southampton Centre” Automatic Urban and Rural Network (AURN) station [49] and the mean value of the sensors of one Air Quality (AQ) Internet of Things (IoT) device at School A and one device at School B, between 1 June and 14 June 2018.

Figure 4 shows a comparison between the “Southampton Centre” Automatic Urban and Rural Network (AURN) station and the mean value of the Air Quality (AQ) Internet of Things (IoT) sensors of one device at School A and one device at School B, between 1 June and 14 June 2018. This graph reveals that the AQ IoT devices are able to capture similar variations and patterns than the AURN station while reporting different values, suggesting that the devices and the AURN station are in relatively similar environments. The differences observed may be due to variations in local environment in which the devices are deployed or to the performances of the sensors. The PM${}_{2.5}$ concentrations measured by the AURN station for the duration of the study have been compared with the measurements of individual sensors from one of the AQ IoT devices located in School A and one in School B. No HPMA115S0 data is available for School B. Table 6 gives the Root Mean Square Error (RMSE) and the Pearson coefficient (r) of the sensor data compared when using “Southampton Centre” monitoring station as a reference, computed using a sensor evaluation toolbox [51].

The Plantower PMS5003 and PMS7003 and the Honeywell HPMA115S0 obtain significantly better RMSE and r than the OPC-N2. The OPC-N2 costs substantially more than the other sensors studied increasing the overall cost of the AQ IoT device and it is not clear that it increases the quality and the accuracy of the measurements.

Each sensor attains similar scores from one site to the other, the results being slightly lower for School B than for School A. This difference between the two schools may simply result from the fact that School B is further away from the reference station and by inter-model variability. A more detailed analysis would be required to properly assess the performances of each sensor, especially through laboratory calibration and collocation with the AURN station; these preliminary indicative results demonstrate that the sensors have a reasonable level of agreement with the “Southampton Centre” monitoring station and that the quality of the data they produce should be further investigated.

Given the overall reliability of the IoT device platform developed and the comparison of the sensors with the reference instruments, the pilot deployment results are encouraging for use in personal PM exposure monitoring.

## 7. Discussion

After installing a total of 24 PM sensors built into six AQ IoT devices across two sites, the entire infrastructure has been evaluated and areas for improvement have been identified. The main focus of the redevelopment effort has been on the hardware, to improve ease of deployment, general flexibility and the ability support up to ten PM sensors.

**Figure 5:**
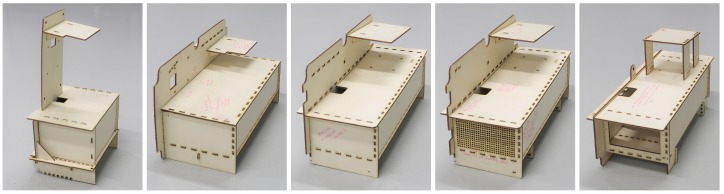
The major changes in the evolution of the Air Quality (AQ) Internet of Things (IoT) interior. Five of twenty-one versions are shown. The final fully populated, acrylic version is shown in Figure 6.

Over 20 design modifications were made to Version 1 of the AQ IoT device. Figure 5 shows the key plywood prototype modifications going from Version 1 to Version 2. Version 2 has three main sections (i) Electronics, (ii) Sensor housing, and (iii) Air intake/exhaust. The electronics section is at the top containing the Raspberry Pi stack, USB Hub and supporting power supplies. The sensor housing is in the middle, providing a reconfigurable area for current and future sensor models. The lower section isolates the air intake from the exhaust air flow. Sensors with an air intake and exhaust on the same side can span the intake/exhaust divider. Other sensor types can exhaust into the sensor housing section and the airflow will be directed out via one of the main air exhausts. Air circulation between the sensor housing and electronics section is kept to a minimum by ensuring that the supporting framework isolates the sections once the enclosure lid is attached. The modifications and their motivation are discussed in the sections below.

### 7.1. Sensors

Future deployments will have the option of including the sensors listed in Table 7, with up to ten PM sensors supported per device. This will support experiments that look at both variance in sensor construction and variance between manufacturers. The Plantower PMS7003 PCB connector board supplied with the sensors has caused intermittent connection issues and needs to be replaced by a direct connection to the sensor pins so this sensor is not currently supported.

### 7.2. Enclosure

**Figure 6:**
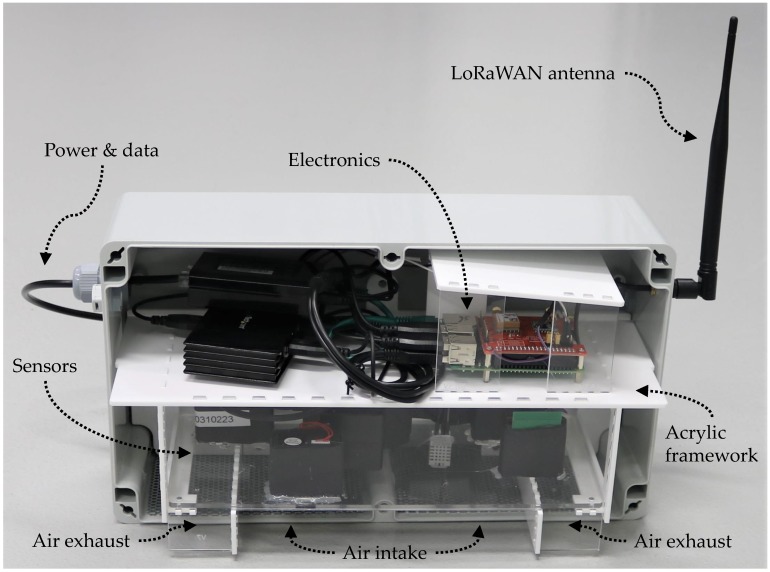
Version 2 of the Air Quality (AQ) Internet of Things (IoT) device in a landscape orientation with the enclosure lid removed. The acrylic framework comprises of (i) the electronics section containing a Raspberry Pi 3, Dragino LoRaWAN Hardware Attached on Top (HAT) and power distribution hardware; (ii) the sensor housing containing five Particulate Matter (PM) sensors, one temperature and humidity sensor and; (iii) the air intake and exhaust, separated by the two vertical acrylic partitions.

The orientation of the enclosure in Version 1 of the AQ IoT device is portrait as shown in Figure 2. In Version 2, the enclosure has been rotated into landscape as shown in Figure 6 and the evolution can be seen in Figure 5. This change provides a larger area for positioning sensors. Version 1 can accommodate up to four PM sensors and Version 2 can accommodate up to ten PM sensors, which allows simultaneous evaluation of an an increased number of sensors, such as those listed in Table 8. Rotating the enclosure results in the Raspberry Pi sitting flat and the on-board GPS antenna having the correct orientation, removing the need for an external GPS antenna.

### 7.3. Modular Build

The AQ IoT device’s plastic enclosure is mounted to the supporting structure, (e.g., a wall) and is not easily removed. This makes hardware maintenance difficult as it has to be carried out in situ, potentially working at height and exposing the electronics to the elements. In Version 2 of the AQ IoT device, the components are mounted on a single modular acrylic framework inside the plastic enclosure, secured by two screws. After disconnecting the antenna and Cat5e cable, the entire framework is removable as a single part, without having to dismount the plastic enclosure from the wall. Component maintenance can then be carried out off-site.

### 7.4. Removable Sensor Board

In Version 1, sensors were glued to the acrylic sensor board which formed part of the framework. Changing the sensor configuration requires up to 20 acrylic pieces to be re-cut and assembled. The sensors need to be glued to the sensor board to ensure there is an airtight seal. In Version 2, the sensor board was made as a single flat removable piece of acrylic. Re-configuring the sensors requires this single sensor board to be re-designed and cut; it is then mounted to the framework using four nuts and bolts.

### 7.5. Manufacturability

Version 2 of the framework has been designed for manufacturability and ease of construction. Each acrylic component either has an obvious orientation, or the orientation is irrelevant. All the framework components for a single sensor device are optimised to be cut out of a single 600 mm × 400 mm × 3 mm (H × W × D) acrylic sheet, with minimal wastage. Multiple framework sensor boards can also be cut from the same sized acrylic sheet, reducing wastage when the sensor configuration is updated.

### 7.6. External Antenna

An external antenna for the LoRaWAN HAT has been added to improve the transmission range. Although the antenna is connected to the side of the enclosure, it is a standard SMA connector, which can support both a stubby antenna or extension cable. This is beneficial where the antenna needs to be higher than the attached building.

### 7.7. Power

In Version 1, the Pi Supply PoE power supply HAT can successfully power a Raspberry Pi 3 model B and four PM sensors—one connected to each of the Raspberry Pi on-board USB ports. Increasing the number of USB ports to ten was achieved by adding a seven port USB hub to one of the Raspberry Pi on-board USB ports. The seven ports on the hub and remaining three on-board USB ports can support a total of ten PM sensors. The PoE HAT has a maximum power output of 6.5 W, which is insufficient to power a Raspberry Pi, ten sensors and a USB hub.

The official Raspberry Pi PoE HAT was tested as a replacement power supply, but it was not able to power the system due to a known hardware bug in the HAT [56]. This hardware bug has since been fixed but was not available in time for incorporation into Version 2. Tests were performed using a PoE splitter outputting a 5 V supply, but the 5 V rail was observed to dip below the 4.63 V under-voltage threshold. When this occurs, the Raspberry Pi reduces the maximum clock-speed to lower the power demands.

The power issue has been overcome by swapping the 5 V PoE splitter with a 12 V output PoE splitter and a total output power budget of 21 W. The 12 V supply is then regulated to produce the required 5 V output. The use of a regulator enables a wider variety of PoE splitters to be used, and means that, if necessary, the load of the sensors can be split across multiple 5 V PSUs.

The on-board USB hub built into the Raspberry Pi supports power cycling of individual USB ports via the uhubctl Linux command [57]. This can be used to save power or restart individual sensors. Given that not all USB hubs support this feature, the USB hub used for Version 2 was carefully chosen to be compatible.

### 7.8. Improved Ventilation

In Version 2, airflow was improved by replacing the grid of 8 mm ventilation holes by two 54 mm intake holes and two 70 mm exhaust holes. Their locations are aligned using a printed template to ensure reproducibility and the four large holes are quicker to fabricate than the grid of smaller holes. The intake and exhaust holes are protected from insects and large debris with the same 3 mm mesh used in Version 1. There is also no longer the need for the exhaust tube, which complicated assembly and maintenance.

Moisture protection for the Raspberry Pi was improved by adding a sloping cover above the main Raspberry Pi stack preventing condensing moisture dripping onto the stack. Two Gore-tex Hylec JDAE12PA7035 [58] breather vents were also added to the electronics section to prevent the build up of condensation.

### 7.9. Software and Sensor Data

The ability to have different sensor configurations for each deployed AQ IoT device adds additional software complexity. Each sensor within the AQ IoT device is sampled by a separate CPU process to limit the impact of hardware and software failures. As the hardware can differ between device, it is necessary to have a configuration file for each device rather than per deployment.

AQ sensor data that is transferred via high bandwidth network connection scales to any sensor configuration as it is file based. The LoRaWAN data bandwidth is limited and can only scale to a few sensors. The current implementation of the LoRaWAN data protocol is optimised to transmit custom data packets, supporting the sensors used in Version 1 of the AQ IoT device. This leads to efficient bandwidth usage but has little flexibility, although the protocol version number can be used to define data packets for the different sensor configurations in Version 2. Dynamically defining the data packets based on the number of sensors is not currently implemented.

### 7.10. Cost

The updated list of components required is shown in Table 7, increasing the cost of a Version 1 style configuration by ≈100 USD. The additional cost offers more flexibility, ease of manufacturing, maintenance and supports a larger volume of PM sensors.

## 8. Future Work

The pilot deployment is still running and the continuous stream of data is being processed and analysed in more detail; this analysis will be the subject of future publications. There are also changes to the hardware and software infrastructure that needs further investigation and are discussed below.

### 8.1. Further Sensor Evaluation

This initial deployment tested four different PM sensors from three different manufacturers; a subset of the available OPC based PM sensors. The sensors for this deployment were chosen based on their popularity in literature, on their ease of use, or the ease of availability. In particular, further work is being conducted to determine (i) how sensor operation varies according to changes in environmental conditions, in the laboratory and in the field, (ii) how sensor data compares in collocation studies, and (iii) whether sensor data from low-cost PM sensors can be calibrated to provide sufficiently reliable data. This is useful where improved spatio-temporal resolution of PM concentrations is required and where reference stations are not viable.

These three points will be evaluated over a period of time with a range of low-cost PM sensors from different manufacturers, as well as identical sensor models. Future field deployments will compare more sensors, enabling a more detailed comparison between manufacturers, cost and variance in manufacturing. While this study provides indicative results regarding the performances of the sensors it cannot firmly conclude on the quality of the data produced without a sensor collocation. As part of this pilot, building a collocation enclosure on top of the “Southampton Centre” AURN monitoring station has been approved and scheduled. All future deployments will be collocated with the AURN monitoring station for an initial period of time, improving the quality of the sensor data.

### 8.2. Wider Scale Deployment

This initial deployment of six AQ IoT devices over two separate sites has shown that useful data can be collected using low-cost OPC based PM sensors. This pilot was appropriate to determine the initial suitability of the data and the reliability of the AQ IoT devices developed. Two Version 2 AQ IoT devices have since been tested during a short three-day field deployment, with encouraging results. After minor modifications, Version 2 will become the basis of future deployments.

The next stage is to increase the number of sensors and locations, in order to achieve high spatio-temporal data resolution. Steps have been undertaken to increase the total number of deployed AQ IoT devices to ≈40 across the city Southampton, an area of ≈50 km^2^.

### 8.3. Operating System

After identifying and implementing all the desired features in the custom Yocto OS, further work is required to ensure that the OS is stable before upgrading the deployed AQ IoT devices. Docker [59] and Singularity [60] have been identified as the two containerisation implementations for all future sensor code deployments, simplifying the OS functionality and improving the management and scalability of deployments.

### 8.4. Data Connectivity

The initial LoRaWAN tests have proven that the technology is suitable for use in urban IoT deployments and all future AQ IoT devices will be LoRaWAN capable. The initial deployment used hard-coded values for the SF and power. The LoRaWAN specification includes Adaptive Data Rate (ADR), which allows a LoRaWAN device to adapt the SF automatically. A limitation of ADR is that the devices have to be stationary for the algorithm to work as it relies on a signal strength average to calculate the SF. As the AQ IoT devices are permanently installed, this limitation does not impose any additional constraints on the existing deployment but is not suitable for future mobile applications.

LoRaWAN allows bi-directional communication. To minimise power consumption the end devices, only listen for downlink messages after they have transmitted an uplink message. Based on the pilot configuration, this introduces downlink latency of up to one hour. The downlink message slots can be used to send control and maintenance messages—for example, to reboot the device or change the sensor configuration.

The system currently uses a custom written data packing scheme to minimise data transfer for each LoRaWAN message; this is not flexible but is bandwidth efficient. Open source libraries for efficient data packing such as Cayenne LPP [61] or Protocol Buffers [62] will be investigated to allow efficient and flexible messages.

Four of the LoRaWAN base stations deployed are Kerlink iBTS devices which support LoRaWAN v2.0 [63]. One of the features offered by LoRaWAN v2.0 is the location of transmitted messages, without the need for GPS. One objective is to make the AQ IoT devices portable and a key limitation is the power requirement. Eliminating GPS and WiFi drastically reduces power requirements, the Raspberry Pi has lower power hardware variants and OS optimisations can assist with reducing overall power consumption.

SF 10 proved a good trade-off between airtime and transmission range, keeping within the duty cycle limits. Table 4 shows that this will facilitate a total payload of 2601 byte/h. By optimising the binary data transmission, selecting only the data channels required, for example PM${}_{2.5}$; hourly data averages; and limiting to a small number of PM sensors, it will be possible to use LoRaWAN to transmit the desired sensor data. This opens up new opportunities for sensor location as the only requirements are power and LoRaWAN coverage. As part of future work, LoRaWAN only devices and alternative power sources are being explored.

## 9. Conclusions

This study has confirmed the feasibility of using low-cost PM sensors to monitor AQ in urban areas. The ongoing larger deployment will investigate further the capabilities of low-cost PM sensors. Four low-cost PM sensors have been selected and deployed in custom-made AQ IoT devices. While differentiating between individual sensor performance is difficult, the deployed AQ IoT devices show that not all PM sensors are equal and that it is possible to achieve a reasonable correlation with the AURN reference stations, with Pearson coefficients ranging from 0.696 to 0.878 and RMSE ranging from 6.065 to 15.756 μg/m^3^.

The AQ IoT device developed is reliable, weather-proof, and capable of supporting PM sensors from different manufacturers. The data from all the AQ IoT devices is retrieved remotely by the pilot platform which can scale to support an arbitrary number of nodes each with a varying number of sensors.

The AQ IoT devices use the LoRaWAN network for communication. With careful selection of hardware and data transmission, it is possible to operate a LoRaWAN based sensor network and remain within the 1% duty cycle. Using LoRaWAN has proven invaluable as a secondary channel of communication, providing hourly readings even during wired network outages and at locations without network access. Although all the raw data is stored on the AQ IoT devices, the LoRaWAN messages confirm device operation, reducing the need for physical site visits.

Following the initial AQ IoT deployment, the device has been modified. The main improvements focus on the redevelopment of the hardware, to improve ease of deployment, general flexibility and the ability support up to ten PM sensors. The new version of the AQ IoT device has been tested during a short field deployment and future developments will be based on this device design.

Further work is being conducted to improve the stability of the OS, to reduce the AQ IoT devices network overheads and to facilitate application updates. Further work is also being undertaken to validate the low-cost PM sensors in controlled laboratory conditions and in the field by deploying a larger network of LoRaWAN enabled AQ IoT devices.

The study concludes that (i) the AQ IoT devices developed can operate at a city scale, (ii) some low-cost PM sensors are viable for monitoring AQ and for detecting PM trends, and (iii) LoRaWAN is suitable for city scale sensor coverage where connectivity is an issue. The findings in this paper show that it is possible to drastically improve the spatio-temporal resolution of PM data at a city scale using low-cost hardware. The underlying datasets for this and previous related publications are available online [47,64].

## Figures and Tables

**Table 1:** Main characteristics of the fan assisted Particulate Matter (PM) sensors used for deployment in Version 1 of the pilot Air Quality (AQ) Internet of Things (IoT) device.

Model	Size (H × W × D) (mm)	Interface	Current Draw @ 5 V DC (mA)	Detection Range (μm)	Upper Limit of Detection (μg/m^3^)	Raw Output
Alphasense OPC-N2 [24]	60 × 64 × 75	SPI	175	0.4 to 17	1500	Yes
Plantower PMS5003 [25]	38 × 21 × 50	UART	100	0.3 to 10	500	Yes
Plantower PMS7003 [26]	37 × 12 × 48	UART	100	0.3 to 10	500	Yes
Honeywell HPMA115S0 [27]	36 × 43 × 24	UART	80	Not known	1000	No

**Table 2:** Comparison of different Low-Power Wide Area Network (LPWAN) technologies [34]. All technologies listed support bi–directional communication, the standard for each technology is driven by different organisations. The different modulation schemes used are Chirp Spread Spectrum (CSS), Binary Phase Shift Keying (BPSK) and Quadrature Phase Shift Keying (QPSK).

	LoRaWAN	Sigfox	NB-IoT
Range (urban)	5 km	10 km	1 km
Range (rural)	20 km	40 km	10 km
Maximum data rate	50 kbit/s	0.1 kbit/s	200 kbit/s
Modulation	CSS	BPSK	QPSK
Encryption	Yes	No	Yes
Adaptive Data Rate (ADR)	Yes	No	No

**Table 3:** List of hardware used to build the Air Quality (AQ) Internet of Things (IoT) device Version 1 at a total cost of ≈900 USD; unit price correct as of November 2018. Each AQ IoT device contains four Particulate Matter (PM) sensors. The costs of laser cutting and labour have not been included.

Item	Description	Quantity	Unit Price (USD)
Raspberry Pi	3 Model B	1	35
Power Over Ethernet (PoE) HAT	Pi supply 83-17278	1	37
Dragino LoRaWAN/GPS HAT	868 MHz	1	36
Micro SD card	Class 10	1	20
Real Time Clock (RTC) module	PiFace RTC Shim	1	10
FTDI USB-Serial Breakout	FT232RL	3	16
USB–USB micro cables	300 mm	3	1
USB–USB A cable	300 mm	1	4
USB–SPI Interface	Robot Electronics USB-ISS	1	32
GPS Antenna	SMA Connector	1	31
Exhaust Pipe	20 mm Conduit	1	2
Steel Mesh	3 mm holes, 51% open area	1	1
Plastic enclosure	Bernstein CT-882	1	51
Laser cut acrylic mounting	—	1	7
Mounting hardware	—	1	25
PoE Injector	Phihong POE31U-1AT-R	1	33
Temperature/Humidity Sensor	DHT22	1	10
Alphasense PM Sensor	OPC-N2	1	443
Plantower PM Sensor	PMS5003	1	18
Plantower PM Sensors	PMS7003	1	18
Honeywell PM Sensor	HPMA115S0	1	33

**Table 4:** LoRaWAN bandwidth per sub–band for Spreading Factors (SF) 7 to 12, calculated at 1% duty cycle, with code rate 4/5 and a 125 kHz bandwidth. Longer distances are achieved with a higher SF which reduces the maximum data transfer. Data Rate and Max Payload from [48]. Other restrictions on usage may lower the available data transfer.

Spreading Factor (SF)	Data Rate (bit/s)	Max Payload (byte)	Max Application Data (byte/h)
SF12	250	51	612
SF11	440	51	1173
SF10	980	51	2601
SF9	1760	115	6095
SF8	3125	222	11,988
SF7	5470	222	21,534

**Table 5:** LoRaWAN base stations located in the city of Southampton, including third party hardware. The Kerlink iBST supports antenna diversity but not all are equipped with dual antenna.

	Name	Altitude (m)	Gateway	Antenna	Third Party
1	7276FFFFFE010292	8	Kerlink iBTS	Procom CXL 900-3LW-NB (Dual)	No
2	7276FFFFFE0103EC	85	Kerlink iBTS	Procom CXL 900-3LW/I	No
3	B827EBFFFEE36EF8	85	IMST iC880A	Procom CXL 900-3LW-NB	No
4	7276FFFFFE0103F0	50	Kerlink iBTS	Procom CXL 900-3LW/IProcom CXL 900-3LW-NB	No
5	B827EBFFFE2D3798	45	IMST iC880A	Taoglas OMB	No
6	B827EBFFFE71AB02	65	IMST iC880A	Taoglas OMB	No
7	B827EBFFFEAC4B12	60	IMST iC880A	RF Solutions FLEXI-SMA90-868	Yes
8	7276FFFFFE01028C	45	Kerlink iBTS	Procom CXL 900-3LW/IProcom CXL 900-3LW-NB	No

**Table 6:** Root Mean Square Error (RMSE) and Pearson coefficient (r) of one Air Quality (AQ) Internet of Things (IoT) device at School A and one device at School B, compared against the “Southampton Centre” Automatic Urban and Rural Network (AURN) station [49].

Sensor	School A		School B	
	RMSE	r	RMSE	r
Alphasense OPC-N2	15.131	0.705	15.756	0.696
Plantower PMS5003	6.065	0.878	6.456	0.850
Plantower PMS7003	6.250	0.875	6.740	0.844
Honeywell HPMA115S0	8.378	0.854	N/A	N/A

**Table 7:** List of hardware used to build the Air Quality (AQ) Internet of Things (IoT) device Version 2 at a total cost of ≈1000 USD for the same sensor configuration as used in Version 1; unit price correct as of November 2018. The costs of laser cutting and labour have not been included. Up to ten separate Particulate Matter (PM) sensors can be attached to a single AQ IoT device. Lines in gray have not changed between Versions 1 and 2.

Item	Description	Quantity	Unit Price (USD)
Raspberry Pi	3 Model B+	1	35
Power Supply Unit (PSU) Splitter	Phihong POE21-120-R	1	45
DC–DC Power Supply Unit (PSU)	5 V 3 A	1	5
Dragino LoRaWAN/GPS HAT	868 MHz	1	36
micro SD card	Class 10	1	20
USB Hub	Dlink DUB-H7	1	41
Real Time Clock (RTC) module	PiFace RTC Shim	1	10
FTDI USB-Serial Breakout	FT232RL	*	16
USB–USB micro cables	300 mm	*	1
USB–USB A cable	300 mm	*	4
USB–DC Jack cable	1 m	1	5
DC Jack Adapter	—	1	1
Cat5e Cable	200 mm	1	1
RF Cable	50 Ω 150 mm	1	3
LoRaWAN Antenna	868 MHz	1	7
USB–SPI Interface	Robot Electronics USB–ISS	*	32
Steel Mesh	3 mm holes, 51% open area	1	1
Plastic enclosure	Bernstein CT-882	1	51
Laser cut acrylic mounting	—	1	7
Mounting hardware	—	1	30
PoE Injector	Phihong POE31U-1AT-R	1	33
Temperature/Humidity Sensor	DHT22	1	10
Alphasense PM Sensor	OPC-N2	*	443
Plantower PM Sensor	PMS5003	*	18
Plantower PM Sensors	PMS7003	*	18
Honeywell PM Sensor	HPMA115S0	*	33
Novafitness PM Sensor	SDS018	*	19
Alphasense PM Sensor	OPC-R1	*	157
Sensirion PM Sensor	SPS30	*	42
Plantower PM Sensor	A003	*	25

* The total component quantity, and final costings depends on PM sensor configuration.

**Table 8:** Main characteristics of the fan assisted Particulate Matter (PM) sensors to be used in Version 2 Air Quality (AQ) Internet of Things (IoT) devices. Each AQ IoT device supports up to ten PM sensors and the configuration can differ between AQ IoT devices.

Model	Size (H × W × D) (mm)	Interface	Current Draw @ 5V DC (mA)	Detection Range (μm)	Upper Limit of Detection (μg/m^3^)	Raw Output
Novafitness SDS018 [52]	59 × 45 × 20	UART	60	0.3 to 10	1000	No
Alphasense OPC-R1 [53]	72 × 22 × 26	SPI	139	0.4 to 12	Not known	Not known
Sensirion SPS30 [54]	41 × 41 × 20	UART I2C	60	0.3 to 10	1000	Yes
Plantower PMSA003 [55]	12 × 35 × 38	UART	100	0.3 to 10	1000	Yes
